# FGOOPNet: A Fuzzy Deep Learning Approach for Mammographic Breast Cancer Classification

**DOI:** 10.2174/0115734056448448260408054729

**Published:** 2026-05-14

**Authors:** Ho Dat Tran, Anh Cang Phan, Thuong Cang Phan

**Affiliations:** 1 Can Tho University, Can Tho, Vietnam; 2 Vinh Long University of Technology Education, Vinh Long, Vietnam

**Keywords:** Breast cancer, Ensemble learning, GOOWE, Fuzzy Gaussian membership, Mammography, Deep learning, CAD systems

## Abstract

**Introduction:**

Breast cancer continues to threaten millions of women globally, and early detection is essential for saving lives. However, inconsistencies in radiological interpretation and uncertainty in lesion assessment limit diagnostic reliability. This study proposes FGOOPNet, a fuzzy deep ensemble model designed to enhance mammographic breast cancer classification accuracy and decision transparency.

**Methods:**

Seven complementary CNN architectures, InceptionV3, ResNet-50V2, ResNet-152, DenseNet-121, DenseNet-201, VGG19, and Xception, were integrated into a unified ensemble framework using the Geometrically Optimum and Online Weighted Ensemble (GOOWE) strategy. GOOWE computes classifier weights through a geometrically optimal least-squares projection, assigning stronger influence to models with lower validation errors. To further refine decision boundaries and quantify predictive uncertainty, a Gaussian-based fuzzy inference module was applied to the ensemble outputs. The proposed system was evaluated on a hybrid dataset combining the public VinDr-Mammo database with an expert-annotated private cohort from regional hospitals, with performance assessed using accuracy, F1-score, and robustness under multiple experimental settings.

**Results:**

FGOOPNet achieved 98.4% accuracy and highly balanced F1-scores for both benign and malignant classes, outperforming individual CNN models and conventional ensemble techniques. The fuzzy inference layer improved reliability in borderline cases by filtering low-confidence predictions and reducing misclassification.

**Discussion:**

The results demonstrate that combining GOOWE weighting with fuzzy Gaussian reasoning enhances both predictive performance and interpretability. This uncertainty-aware mechanism helps address limitations in conventional CAD systems, particularly in heterogeneous breast tissue and visually ambiguous regions.

**Conclusion:**

FGOOPNet offers a robust and explainable solution for mammographic breast cancer classification, highlighting its potential for integration into clinical decision-support workflows. Future work will focus on extending the model to multi-modal imaging and large-scale multi-center validation.

## 1 INTRODUCTION

Breast cancer remains the most frequently diagnosed cancer among women worldwide and continues to be one of the leading causes of cancer-related mortality [1], accounting for substantial public health and socioeconomic burdens. According to global cancer statistics, early detection and timely intervention significantly improve patient prognosis and survival rates. Mammography evolved from early X-ray breast imaging in the 1950s, with major technological milestones including screen-film systems, Full-Field Digital Mammography (FFDM), and Computer-Aided Detection (CAD). These developments paved the way for current AI-assisted diagnostic systems. It enables the visualization of microcalcifications, architectural distortions, and subtle masses that may be indicative of malignancy at an early, more treatable stage. However, mammogram interpretation is inherently challenging due to factors such as inter-observer variability, differences in radiologists’ expertise, and the intrinsic heterogeneity of breast tissue, particularly in women with dense breasts. These challenges often lead to false positives, unnecessary biopsies, or missed diagnoses, thereby underscoring the need for supportive diagnostic tools. In response to these limitations, Computer-Aided Diagnosis (CAD) systems have been developed to assist radiologists by providing consistent, objective, and reproducible image analyses. Early CAD systems relied heavily on handcrafted features, such as texture descriptors, shape parameters, and intensity-based metrics, combined with traditional classifiers, including support vector machines, decision trees, and k-nearest neighbors. While such methods achieved moderate success, they were limited by their inability to adapt to the complex, high-dimensional patterns inherent in mammographic images. The advent of deep learning, and in particular Convolutional Neural Networks (CNNs), has revolutionized medical image analysis by enabling end-to-end learning of hierarchical features directly from raw image data. CNN-based approaches have demonstrated remarkable performance in detecting and classifying breast lesions, often achieving results comparable to or surpassing those of experienced radiologists in controlled experimental settings [2]. Architectures such as Inception, ResNet, DenseNet, VGG, Xception, and EfficientNet have been successfully adapted to mammogram analysis, leveraging their capacity to extract multi-scale features, reduce vanishing gradients, and optimize computational efficiency. Advances such as transfer learning, domain adaptation, and attention mechanisms have further enhanced model generalizability, especially in scenarios where annotated medical datasets are limited. Furthermore, the integration of segmentation networks like U-Net or Mask R-CNN has facilitated precise lesion localization, thereby augmenting the interpretability of classification decisions. Despite these advances, reliance on a single deep learning model can lead to overfitting and suboptimal robustness, particularly when confronted with data from different imaging devices, acquisition protocols, or patient demographics [3]. Ensemble learning, which combines multiple models to exploit their complementary strengths, offers a robust framework for improving predictive accuracy, reducing variance, and enhancing generalization [4, 5]. By aggregating diverse predictive patterns, ensemble methods mitigate the limitations of individual models and provide a more reliable foundation for real-world clinical deployment in breast cancer screening and diagnosis.

## 2 RELATED WORK

Recent studies have explored a wide range of ensemble and deep learning approaches to improve breast cancer classification performance across different datasets and imaging modalities. Traditional ensemble methods, such as the majority voting system developed by Dhas *et al*. [6] combined SVM, KNN, and Random Forest, achieving 96.5% accuracy on the WBCD dataset; however, the model exhibited sensitivity to class imbalance and lacked adaptability to evolving data streams. Hybrid deep learning approaches have also shown promise. Yasser [7] proposed the BCDNet–VGG16 hybrid model, reaching 98.7% accuracy on BreakHis, although effectiveness remained highly dependent on handcrafted feature extraction. Data augmentation–driven improvements were demonstrated by Kaffashbashi *et al*. [8], who obtained 95.4% accuracy on MIAS through enhanced generalization strategies, albeit at the cost of increased preprocessing complexity.

EfficientNet-based architectures have received significant attention for their lightweight yet high-performing design. Behzadpour *et al*. [9] achieved 97.2% accuracy on DDSM using EfficientNet-B0, while Lilhore *et al*. [10] enhanced diagnostic performance on BreakHis to 98.4% by integrating EfficientNet with Bi-LSTM networks. Despite these advances, limitations such as overfitting, dependency on single datasets, and high computational burdens remain. Additional ensemble strategies have been proposed, such as the phase-based deep ensembles of Molina *et al*. [11], which reached 96.1% accuracy on INbreast but lacked online learning capability and real-time adaptability. Classic ensemble learning frameworks from Jabbar [12] and Hosni *et al*. [13] highlighted the benefits of model diversity yet did not incorporate dynamic weighting or streaming adaptation. Other predictive ensemble models, such as those by Nagarajan *et al*. [14] and Mahesh *et al*. [15], demonstrated accuracies of 92.8% and 94.2%, respectively, though their performance degraded in noisy or imbalanced conditions. Online ensemble learners like the Hoeffding Tree–Naïve Bayes hybrid of Alhayali *et al*. [16] achieved 91.6% on streaming MOA datasets but struggled with nonlinear decision boundaries, while adaptive-voting frameworks such as Batool *et al*. [17] required substantial manual tuning despite achieving 97.5% accuracy.

More recent deep ensemble and meta-learning frameworks also contributed to performance improvements. Ali *et al*. [18] demonstrated a CNN-based meta-learning ensemble that attained 98.3% accuracy but suffered from limited interpretability and computational complexity. Similarly, Das *et al*. [19] produced 96.8% accuracy on DDSM using a deep ensemble framework, but required full retraining when encountering new data, restricting real-time applicability. A significant contribution to online ensemble learning is the GOOWE method by Bonab *et al*. [5], which geometrically optimizes classifier weights for evolving data streams; however, it does not explicitly manage uncertainty or fuzziness present in medical imaging environments.

Recent advances from 2023 to 2025 further highlight ongoing challenges and research gaps. Qasrawi *et al*. [20] developed a hybrid ensemble integrating YOLOv5, MedSAM, and a functional-link neural network, achieving up to 99.7% accuracy on a large clinical mammography dataset, though its generalizability remained limited due to single-center data and the lack of uncertainty modeling. Shah *et al*. [21] fine-tuned multiple CNN backbones and ensemble them on CBIS-DDSM, reaching 85% accuracy, but without addressing model calibration or interpretability. Chegini and Mahlooji Far [22] introduced an uncertainty-aware CAD system combining mammography and ultrasound with probabilistic deep learning, achieving AUC values above 0.90, yet constrained by modest dataset sizes. Multimodal fusion strategies were also explored: Chen *et al*. [23] fused mammography and ultrasound using deep CNNs, attaining 93.78% accuracy and AUC 0.968 on a 790-patient cohort, although the system remained two-class and single-center. Multi-stage architectures such as the MSDLM framework by Ahmad *et al*. [24] integrated Gaussian U-Net segmentation with EfficientNet-based classification, achieving 97.6% accuracy on CBIS-DDSM but without any fuzzy reasoning or adaptive weighting. Robust ensemble refinements were proposed by Shinde and Kulkarni [25], who combined DenseNet, InceptionV3, and EfficientNetB0 with focal-loss tuning to reach 99% accuracy on INbreast, while still lacking uncertainty modeling. A fuzzy-rank-based CNN ensemble by Deb and Jha [26] showed improved robustness on BUSI ultrasound images, although it was limited to ultrasound and did not generalize to full-field mammography.

Collectively, these studies demonstrate the effectiveness of ensemble and deep learning strategies but also reveal persistent gaps: most models lack online adaptability, semantic interpretability, uncertainty handling, and robust performance under distribution shifts. To address these limitations, this study introduces a Robust Hybrid Fuzzy-Gaussian GOOWE Ensemble for mammographic breast cancer classification, integrating fuzzy inference with online geometric weighting to enhance adaptability, interpretability, and robustness in real-world clinical environments. The main contributions of this paper are:

- Optimized online weighted ensemble classification using the GOOWE strategy, which dynamically adjusts classifier weights in real-time to enhance breast cancer prediction from mammograms;
- Integration of fuzzy Gaussian inference with GOOWE, enabling semantic interpretability and improved decision transparency, especially in borderline or ambiguous cases;
- Development of a fuzzy threshold mechanism that enhances classification accuracy by refining decision boundaries based on uncertainty modeling;
- Parametric analysis and tuning of Gaussian membership functions, ensuring the inference system is well-adapted to the distribution characteristics of mammographic features;
- Construction of a comprehensive hybrid dataset combining the public VinDr-Mammo dataset and an expert-labeled dataset collected from regional hospitals, providing a rich and clinically relevant data source for model training and evaluation.

These enhancements not only improve classification performance and interpretability but also lay the foundation for real-world deployment in dynamic and uncertain clinical environments. Table **1** summarizes the notation and mathematical symbols used throughout the paper, providing a consistent reference for the proposed methodology.

## 3 BACKGROUND

### 3.1 Deep Learning

Computer-Aided Diagnosis (CAD) systems employ computers to aid medical diagnosticians in making decisions. CAD systems are widely utilized in medical imaging, including X-rays, Computed Tomography (CT), and Magnetic Resonance Imaging (MRI), to detect anomalies associated with illnesses. Traditional CAD algorithms use image processing techniques to detect suspicious regions. These systems frequently rely on a sophisticated collection of handmade properties, such as texture, shape, and gray-level intensity, which human specialists must extract over time. Traditional computer-aided detection may diagnose breast cancer with high accuracy, particularly when current machine learning techniques are utilized. Traditional CAD systems, which rely on hand-crafted features, cannot handle complex changes in tumor form and density distribution, resulting in a significant proportion of false positives. To address the issue of hand-crafted features in traditional CAD, several recent studies have applied sophisticated Deep Learning models at various stages of CAD systems. Deep learning models may learn and extract high-level features from raw input data, resulting in dramatically improved classification performance over earlier techniques.

Deep learning, a form of artificial intelligence, has emerged as an important technique in medical image segmentation due to its ability to automatically learn and extract features from complex data. Unlike traditional approaches that rely on hand-crafted features, deep learning analyzes images using deep neural networks, which are particularly successful in segmentation tasks such as detecting tumors, organs, or lesions in CT, MRI, or X-ray images. Deep learning network models, such as InceptionV3 [27], DenseNet, ResNet, VGG19, and Xception, are important for medical picture classification and segmentation because they extract complex and efficient features. InceptionV3, with its parallel Inception blocks and convolutional decomposition techniques, increases processing efficiency while maintaining excellent accuracy in classification applications such as skin cancer diagnosis. DenseNet-121 and DenseNet201 employ dense connections to increase information transmission, decrease gradient loss, and are successful in chest X-ray or mammography classification [28]. ResNet-152 and ResNet-50V2, with skip connections, allow for deep network training while maintaining accuracy and are commonly used in MRI or CT analysis [29]. VGG19's fundamental architecture and 3x3 kernel make it appropriate for transfer learning in skin lesion or X-ray classification. Xception, based on depth-separated convolution, reduces parameters while increasing speed. It is used for COVID-19 and breast cancer diagnosis [30]. When trained on large datasets such as ImageNet or specialized medical data, these models perform similarly to or better than human physicians in a number of tasks.

U-Net [31] is a popular segmentation model with an encoder-decoder architecture. It is highly valuable in medical image segmentation because of its capacity to combine both global and local data. Fully Convolutional Networks (FCN) allow for pixel-by-pixel segmentation without the necessity of a fully connected layer, and sophisticated models such as DeepLab, which use atrous convolution to broaden the field of view while maintaining high resolution.

The Facebook AI Research (FAIR) team created Mask R-CNN [32], an advanced deep learning model, in 2017. It aims to address the problem of instance segmentation in computer vision. This model builds on Faster R-CNN by including a mask prediction branch for each item, which runs alongside the classification and bounding box regression branches. Mask R-CNN's distinguishing characteristic is its capacity to create pixel-accurate segmentation masks for each detected item. This is made possible by the ROIAlign methodology, which enhances accuracy when compared to traditional ROI Pooling techniques. Mask R-CNN has outperformed on a number of benchmark datasets, including COCO. It is used in a broad range of applications, including autonomous cars, medicine, robotics, and document text recognition. Furthermore, this model serves as the foundation for other current systems, like Detectron2, and it continues to play a key role in ongoing computer vision research.

Furthermore, data augmentation, transfer learning, and attention methods are used to improve model performance on small and heterogeneous medical datasets. These developments have improved accuracy in applications such as brain, heart, and lung tumor segmentation, helping clinicians to better diagnose and plan treatments while also opening the path for automated medical systems.

### 3.2 Fuzzy Logic

**Fig. (1):**
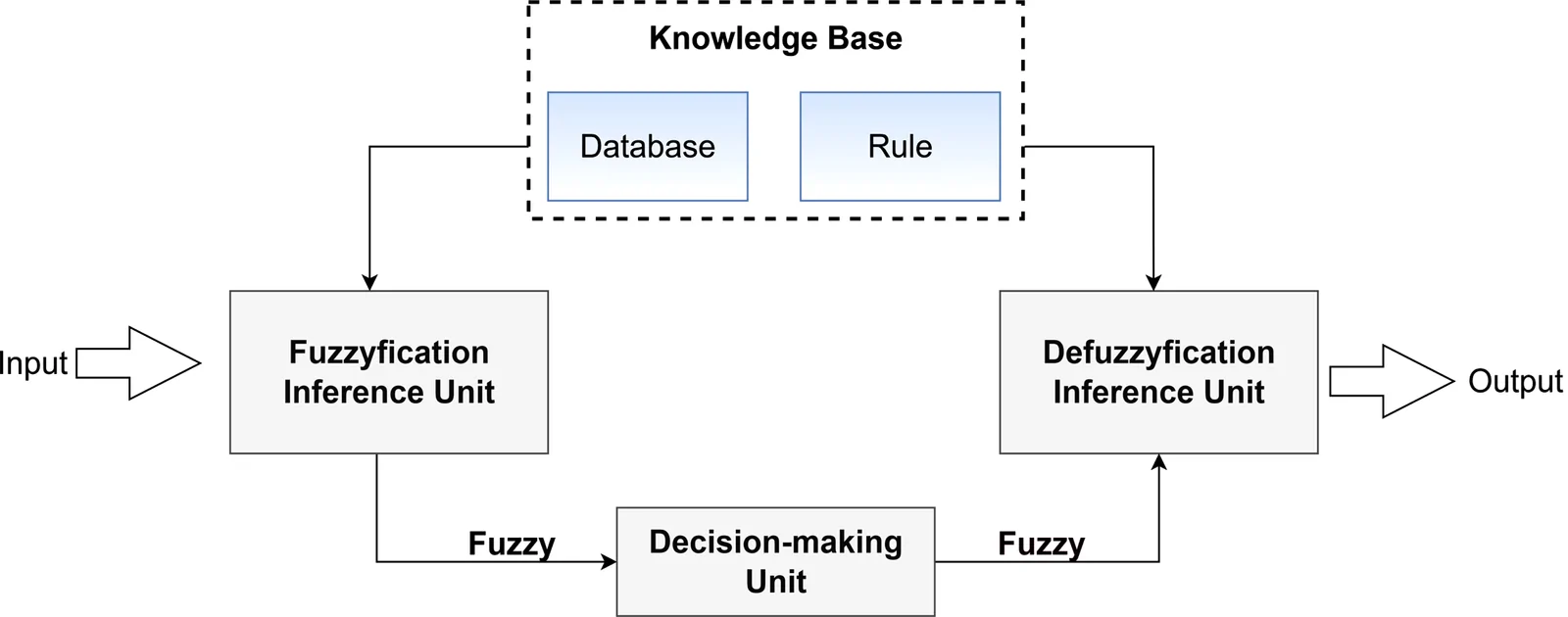
Fuzzy Inference System (FIS) [33].

Fuzzy logic is an approach used to handle information and make decisions in situations involving vagueness or uncertainty. In contrast to traditional logic, which operates with strict binary values (0 or 1), fuzzy logic introduces a continuum of values between 0 and 1, allowing it to capture partial truths [33]. Fuzzy machine learning integrates these fuzzy principles into machine learning algorithms, making them particularly suitable for analyzing uncertain or imprecise data, such as that found in medical imaging. These models help interpret and identify abnormal image regions more effectively, enhancing diagnostic accuracy and classification performance. A fuzzy inference system facilitates decision-making through a structured set of fuzzy rules and a comprehensive knowledge base (Fig. **1**).

- Input (Crisp Data Acquisition): The system begins with the acquisition of crisp (precise numerical) input values representing measurable features or variables of the problem domain. These values are often obtained from sensors, measurements, or preprocessed data sources. At this stage, the data exists in its original, non-fuzzy form and cannot yet be directly processed by fuzzy logic operations.
- Fuzzification Inference Unit: The fuzzification stage transforms crisp input values into fuzzy sets by evaluating them against predefined membership functions. Each membership function represents a linguistic term (*e.g*., “low,” “medium,” “high”) within the variable’s domain. The fuzzification process assigns a degree of membership between 0 and 1 to each input for all relevant fuzzy sets. This step enables the system to handle uncertainty and partial truth, allowing inputs to belong to multiple sets simultaneously with varying degrees.
- Knowledge Base: Database and Rule Base: Database: Contains the definitions of membership functions for all fuzzy variables, including their shapes (*e.g*., triangular, trapezoidal, Gaussian) and parameters. Rule Base: Consists of a collection of fuzzy IF–THEN rules that capture expert knowledge or system behavior.
- Decision-Making Unit (Inference Engine): The inference engine applies the fuzzy rules to the fuzzified inputs to determine the fuzzy output sets. Using logical operators such as fuzzy AND (minimum), OR (maximum), and implication methods (*e.g*., Mamdani or Sugeno), the engine combines the contributions of all rules. This results in one or more aggregated fuzzy output sets representing the system’s reasoning in fuzzy space.
- Defuzzification Inference Unit: Since real-world actuators or decision processes require crisp values, the aggregated fuzzy output must be converted back into a single crisp output. Defuzzification applies mathematical techniques such as the centroid (center of gravity), bisector, or mean of maxima methods to compute a precise value that best represents the fuzzy output set.
- Output (Crisp Decision/Action): The final crisp output represents the system’s decision, recommendation, or control action. It is ready for direct interpretation, further processing, or execution in the target application.

By allowing inputs and outputs to be expressed in linguistic terms and handled as fuzzy sets, the system accommodates uncertainty, nonlinearity, and vagueness inherent in many real-world problems. This makes FIS particularly valuable in control systems, medical diagnosis, decision support systems, and pattern recognition.

### 3.3 Ensemble Learning

Ensemble Learning is a valuable machine learning technique in which numerous separate models are integrated to form a more powerful ensemble model. The primary idea behind this strategy is to use the diversity of models to compensate for each other's faults, boosting the system's accuracy and generalization. Ensemble learning is very significant in deep learning because it reduces overfitting, increases prediction stability, and improves performance on complicated datasets, notably in medical settings where data is frequently heterogeneous, noisy, and difficult to acquire. Studies have demonstrated that ensembles of many deep learning models frequently outperform single models by using each model's specific strengths. Model ensemble approaches can be classed based on the level of aggregation in the machine learning process. There are three basic levels:

- Data-level ensemble: At this level, the component models are trained on multiple datasets or processed parts of the data (for example, partitioning the training set and using various data augmentation techniques). The goal is to improve the diversity of the models so that when they are joined, the total system becomes more stable and accurate. Bagging methods, such as Random Forest, are a classic example.
- Feature-level ensemble: In this strategy, aggregation occurs during the feature representation stage. Deep learning models can be used to extract various features from data, which are then combined or aggregated before being fed into the final classification model. This strategy is frequently utilized in multimodal systems, such as merging medical pictures and clinical data.
- Output-level ensemble: The most typical strategy in deep learning is to aggregate each model's output predictions to obtain a final result. Common strategies include majority voting, weighted voting, and stacking (using a meta-model to learn from component model outputs). Output-level ensembles frequently enhance accuracy without changing any model's internal architecture.

### 3.4 Geometrically Optimum and Online Weighted Ensemble (GOOWE)

The GOOWE (Geometrically Optimum and Online Weighted Ensemble) method is developed to improve the classification performance of deep learning systems by determining the linear combination of component models to optimize accuracy. Unlike basic ensemble methods such as Hard Voting or Soft Voting, which treat models as equal or assign fixed weights, GOOWE determines optimal geometric weights that reflect the relationship between model outputs and the actual label. The core of GOOWE is to evaluate the difference between the prediction of each model and the actual label to determine which model contributes more to the final result. The model with higher accuracy will be assigned a larger weight in the ensemble process, while the weaker model can be retained for diversity or eliminated if it is not useful. A key advantage of GOOWE is that it does not require the base classifiers to share the same architecture or training dataset, thus being flexible in combining many different deep learning models, such as CNN, ResNet, DenseNet, or Inception.

Let the outputs of the classifiers be represented as probability vectors s_i_ = [s_i1_, s_i2_,..., s_iK_] for each classifier C_i_ with *K* classes. The goal is to find the optimal weights w_i_ for each classifier so that the linear combination of predicted outputs is closest to the true label. The objective function is described by the squared error function given by the formula Eq. (**1**):


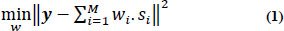


Where *y* is the one-hot actual label vector, s_i_ is the probability vector of the classifier C_i_, w = [w_1_, w_2_,..., w_M_] is the weight vector to be optimized, *M* is the number of classifiers. Additionally, the Gaussian membership function used in this paper is defined in Eq. (**2**):


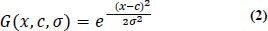


Where, x: input value, c: mean of Gaussian function – maximum value point, *σ*: standard deviation – controls the width of the curve, G(x) *ϵ* (0,^1^]: the value of the function is always in the range (0, 1), reaching the value 1 at x=c.

### 3.5 Evaluation Metric

Precision, Recall, and F1-score metrics are commonly employed to assess the object detection model. These measurements are computed using the following equations: Precision is defined as the ratio of True Positives (TP) to the total number of predicted positive cases, where False Positives (FP) represent incorrect predictions, as delineated in Eq. (**3**). Recall, or sensitivity, is calculated as the ratio of True Positives (TP) to the total number of actual positive cases, with False Negatives (FN) denoting missed predictions, as specified in Eq. (**4**). Given that Recall and Precision are distinct concepts, the F1_score Eq. (**5**) serves as an indicator to assess the average of Precision and Recall.


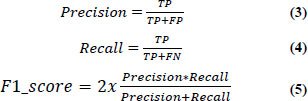


To evaluate the average precision across all classes, we use the mAP, which is calculated using Eq. (**6**).


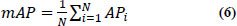


## 4 METHODOLOGY

The content of the study presents a method for classifying breast cancer on mammograms based on the GOOWE weighted optimal combination model combined with Gaussian fuzzy inference. The method includes two main phases: the training phase and the testing phase. The phases of the method are presented in the Fig. (**2**).

### 4.1 Training Phase

Data preprocessing: The input mammograms are enhanced using contrast enhancement and noise removal techniques to highlight histopathological features. This is followed by data augmentation, such as flipping, rotating, and adjusting brightness, to increase the diversity of the training set and reduce the risk of overfitting.

Feature extraction using Deep Neural Network: Image features are extracted using network architectures such as InceptionV3, ResNet-50V2, ResNet-152, DenseNet-121, DenseNet-201, VGG19, and Xception. The outputs of these networks are fed into a fully-connected layer and a sigmoid activation function to represent the predicted probability.

Model training: The results from deep learning models are stored as trained models, which are used to extract features in the testing phase.

### 4.2 Testing Phase

Preprocessing and feature extraction: The new mammogram image is processed similarly to the training phase, then features are extracted by reusing the trained deep learning models.

Classification using GOOPNet and FGOOPNet: The extracted features are fed into the ensemble classifier. In which GOOWE is used to calculate the optimal weight for each component model based on the geometric projection technique. Next, the Fuzzy GOOWE model applies a Gaussian fuzzy inference system to evaluate the certainty level of each classification result, which helps to handle edge and unclear data cases well.

Let M_i_ denote the i^th^ deep learning model selected from a pool of seven pre-trained architectures, including InceptionV3, ResNet-50V2, ResNet-152, DenseNet-121, DenseNet-201, VGG19, and Xception. Given a mammographic image *x*, the goal is to predict its class label 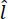, where the ground truth label *ℓ* ϵ {0, 1} represents benign (0) or malignant (1). Each model M_i_ produces a class probability P_i_(x) ϵ [0, 1], indicating the likelihood of *x* being malignant. The prediction scores from the top-3 most accurate models are collected into a matrix *A*, and a weight vector w = [w_1_, w_2_, w_3_] is computed using geometric projection and least squares optimization to minimize the error between predicted and actual labels. The ensemble score is calculated as s(x) = w_1_P_1_(*x*) + w_2_P_2_(*x*) + w_3_P_3_(*x*), and a threshold-based classification is applied.

To improve decision reliability, a fuzzy inference module based on Gaussian membership functions is integrated. Specifically, the membership degrees *µ*_0 _(*x*) and *µ*_1_(*x*) quantify how strongly the image belongs to the benign or malignant class, respectively. These values are computed using Gaussian functions centered at 0 and 1 with a standard deviation *σ* = 0.2. A fuzzy confidence threshold τ_*fuz*_ = 0.45 is used to filter out uncertain predictions. The overall input to the system includes a labeled training set, an unlabeled test set, model outputs, and fuzzy parameters. The final outputs consist of the predicted label and a confidence score indicating the reliability of the classification decision.

Based on Algorithm **1**, the proposed method is extended by integrating a Gaussian fuzzy inference component to improve decision-making ability, especially in uncertain classification cases. Instead of relying solely on the output probability value to classify images as benign or malignant, the model calculates the suitability (also known as “confidence”) of the sample for each class. This approach helps the system avoid making decisions in uncertain situations, thereby improving the overall reliability and accuracy of the classification model.

**Algorithm 1:** GOOPNet algorithm.

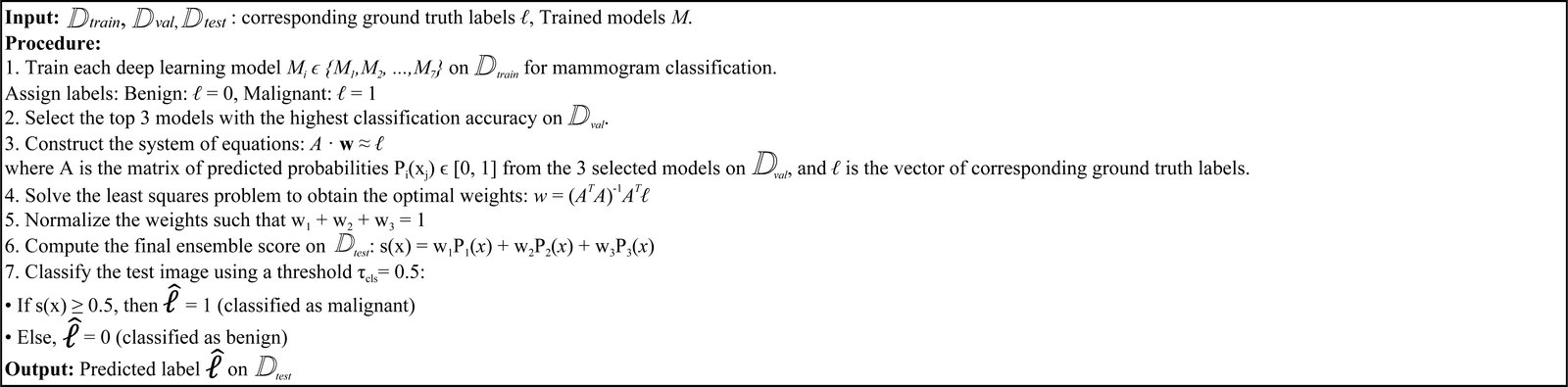

This method uses the three most accurate models out of the seven original models through the training process. The ensemble is only performed on the strongest models to ensure that the input to the fuzzy inference system is more reliable and stable.

Integrating the Gaussian fuzzy inference component into the GOOWE weighted ensemble model enhances the classification process's reliability. Not only does the model rely solely on the highest probability to produce results, but it can also evaluate the certainty of each prediction using two key parameters: *σ* and τ_*fuz*_. The fuzzy Gaussian inference layer receives the GOOWE ensemble score as input and computes membership degrees to benign and malignant classes using Gaussian functions centered at 0 and 1. Uncertainty is quantified by the peak membership response, and low-confidence scores are filtered using a reliability threshold before final classification. The details are presented in Algorithm **2**.

**Algorithm 2:** FGOOPNet algorithm.

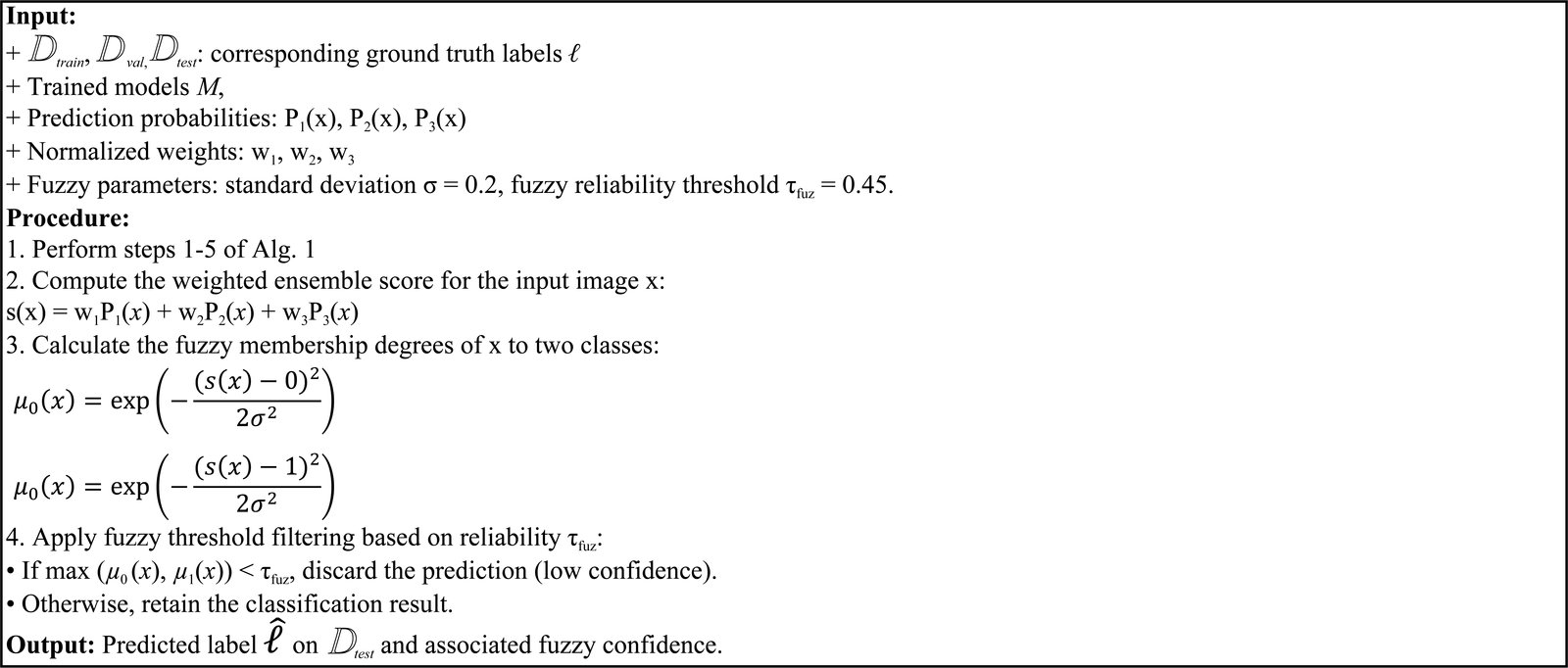

The fuzzy inference system may be further extended by incorporating additional linguistic rules (*e.g*., ‘slightly malignant’, ‘highly suspicious’) or by learning membership parameters adaptively. This enhancement can provide more granular confidence patterns and clearer human-interpretable explanations to radiologists, especially in borderline mammographic cases.

## 5 EXPERIMENTAL RESULTS

### 5.1 Applicable Scenarios

For the proposed model, we conducted experiments across nine scenarios using the training parameters described in Table **2**.

### 5.2 Installation Environment and Dataset

This study examines mammography pictures with masses from two datasets to assess the efficacy of the proposed algorithms. To evaluate the model's efficacy in a domestic setting, we employ the VinDr-Mammo [34] dataset, comprising 20,000 pictures from 5,000 cases obtained from four conventional mammographic views (CC and MLO). This dataset includes BI-RADS classifications from 1 to 5 and bounding box annotations for lesion areas.

All datasets utilized in this research are either publicly accessible or gathered with appropriate ethical authorization. VinDr-Mammo is an open-access dataset widely used within the medical imaging community. This dataset complies with the data usage regulations of the original source. Furthermore, we acquired a private dataset from local hospitals, comprising 1,045 photos that were classified and annotated by medical professionals. This dataset was anonymised and collected with the agreement of local medical ethics boards, guaranteeing adherence to all relevant rules. To prevent data leakage, all patient-level images were grouped by patient ID before splitting. The dataset was divided into 70% training, 10% validation, and 20% testing with no cross-patient overlap (Table **3**). All augmentations were applied only to the training set. The system functions within a Python environment on Kaggle, utilizing an Intel Xeon 2.00 GHz processor and 30GB of RAM.

Based on the results in Fig. (**3**), the Acc and Loss measures over the epochs of the 7 scenarios tend to increase in accuracy and decrease in loss steadily over each epoch. Most scenarios achieved high accuracy and low loss values, indicating effective learning and good convergence. Scenario 3 exhibits fluctuations and discrepancies between training and validation, leading to low output performance.

### 5.3 Training Results

**Fig. (4):**
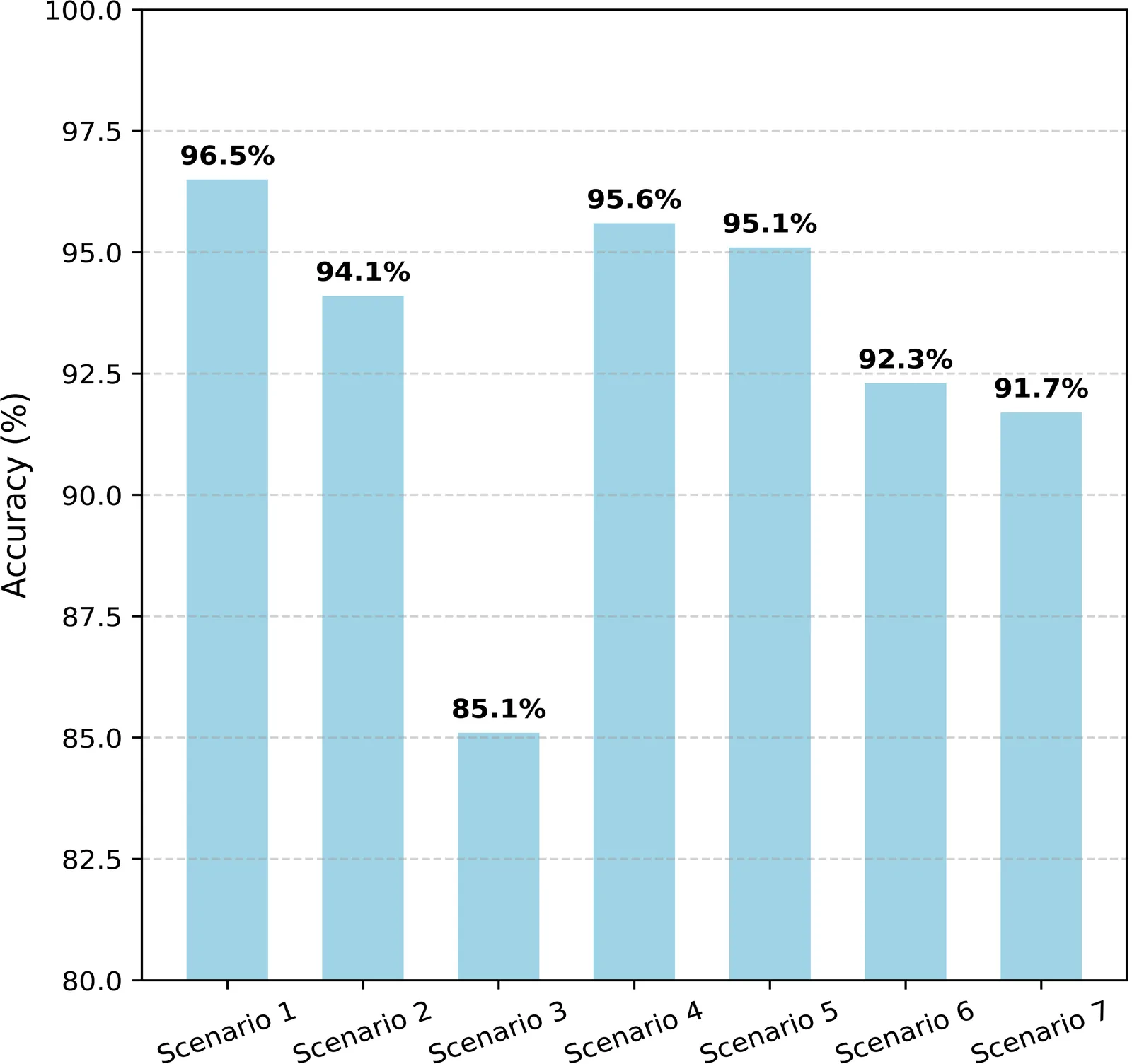
Accuracy comparison chart between trained models.

Figure **4** shows the classification performance of each model on the test dataset. In particular, Sce. 1 achieved the highest Accuracy with 96.5%, followed by Sce. 4 (95.6%) and Sce. 5 (95.1%). These models demonstrate the ability to learn effective features for distinguishing between benign and malignant tumors in mammograms, enabling accurate classification. In addition, despite the longer training time, Sce. 3 only achieved Accuracy 85.1%, the lowest among the compared models. This indicates limited generalization capability, potentially due to architectural limitations when applied to mammographic patterns. The remaining scenarios, such as Sce. 2 (94.1%), Sce. 7 (91.7%), and Sce. 6 (92.3%), have fairly good performance and will be suitable choices for the model combination process. In addition to accuracy, we compare the loss measure, which quantifies the deviation between the predicted and actual labels. The models with lower loss have closer predictions to reality, indicating that the models learn better.

**Fig. (5):**
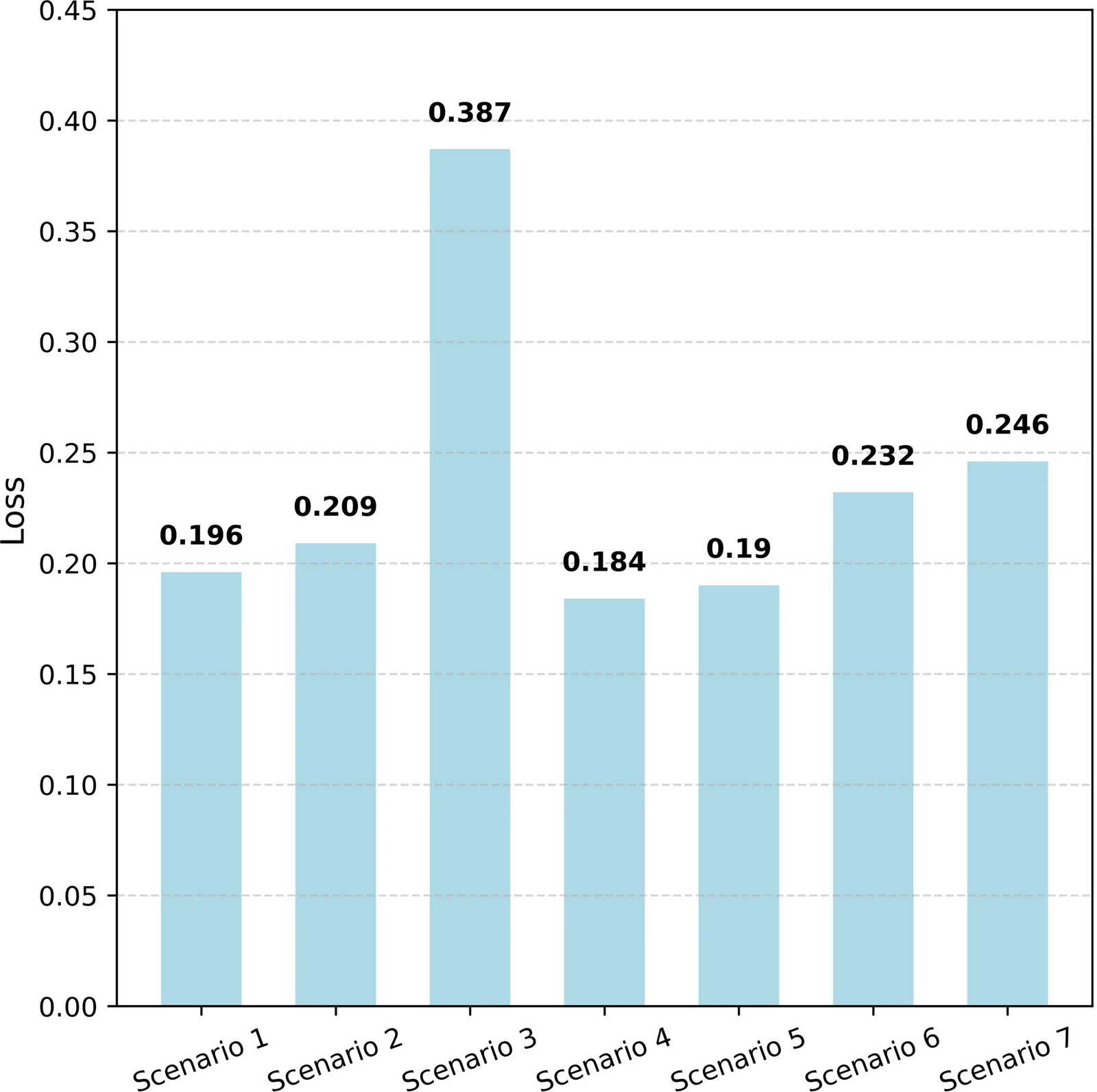
Loss comparison chart between trained models.

Figure **5** shows the loss values of different deep learning models during training, showing clear differences between the architectures. Sce. 4 achieved the lowest loss (0.184), indicating effective learning and good generalization, despite being a deep and complex model. Sce. 1 and Sce. 5 also performed quite well with losses of 0.196 and 0.190, respectively, demonstrating a good balance between performance and model complexity. Meanwhile, Sce. 3 had the highest loss (0.387), which may reflect overfitting or difficulty in training due to the dense structure of the model. Sce. 2 performed much better than Sce. 3, despite being a lighter architecture, showing that increasing the model depth does not always result in better results. Finally, Sce. 6 and Sce. 7 have losses of 0.232 and 0.246, respectively, which are in the medium range, reflecting stability but not optimization compared to the ResNet or Inception models. Overall, the ResNet models show superior performance on the mammography dataset, especially when considering the relationship between depth, complexity, and loss value.

The training time results of the proposed models are summarized in Fig. (**6**), reflecting the difference in time between the models. Among them, Sce. 3 takes the longest training time with 156.52 minutes, followed by Sce .6 with 136.75 minutes and Sce. 4 with 81.63 minutes. This clearly reflects the relationship between network depth, architectural complexity, and computational cost during training. In contrast, models such as Sce. 5 (52.27 minutes) and Sce. 2 (67.38 minutes) have significantly shorter training times, thanks to their more compact and optimized architectures. This result shows that, in applications that require practical deployment or limited resources, models with short training times may be a suitable choice. Training time analysis plays an important role in considering model selection, ensuring a balance between classification efficiency and computational cost when applied in practice.

Threshold (T) acts as a threshold to determine the confidence level of the prediction. When the difference between the two classes (benign and malignant) is less than T, the model will consider it as an “uncertain” prediction, meaning that it does not clearly distinguish between the two classes. These cases are still retained but are recorded as having a low confidence level and should be considered more carefully during the evaluation process. In this method, the classification threshold is applied by evaluating the F1-score value corresponding to each threshold level to find the optimal point. The results show that the F1-score reaches its maximum at a threshold of 0.45, which is a suitable choice for balancing accuracy and sensitivity. Choosing this threshold helps improve confidence in the prediction, especially when classifying samples with unclear characteristics.

**Fig. (7):**
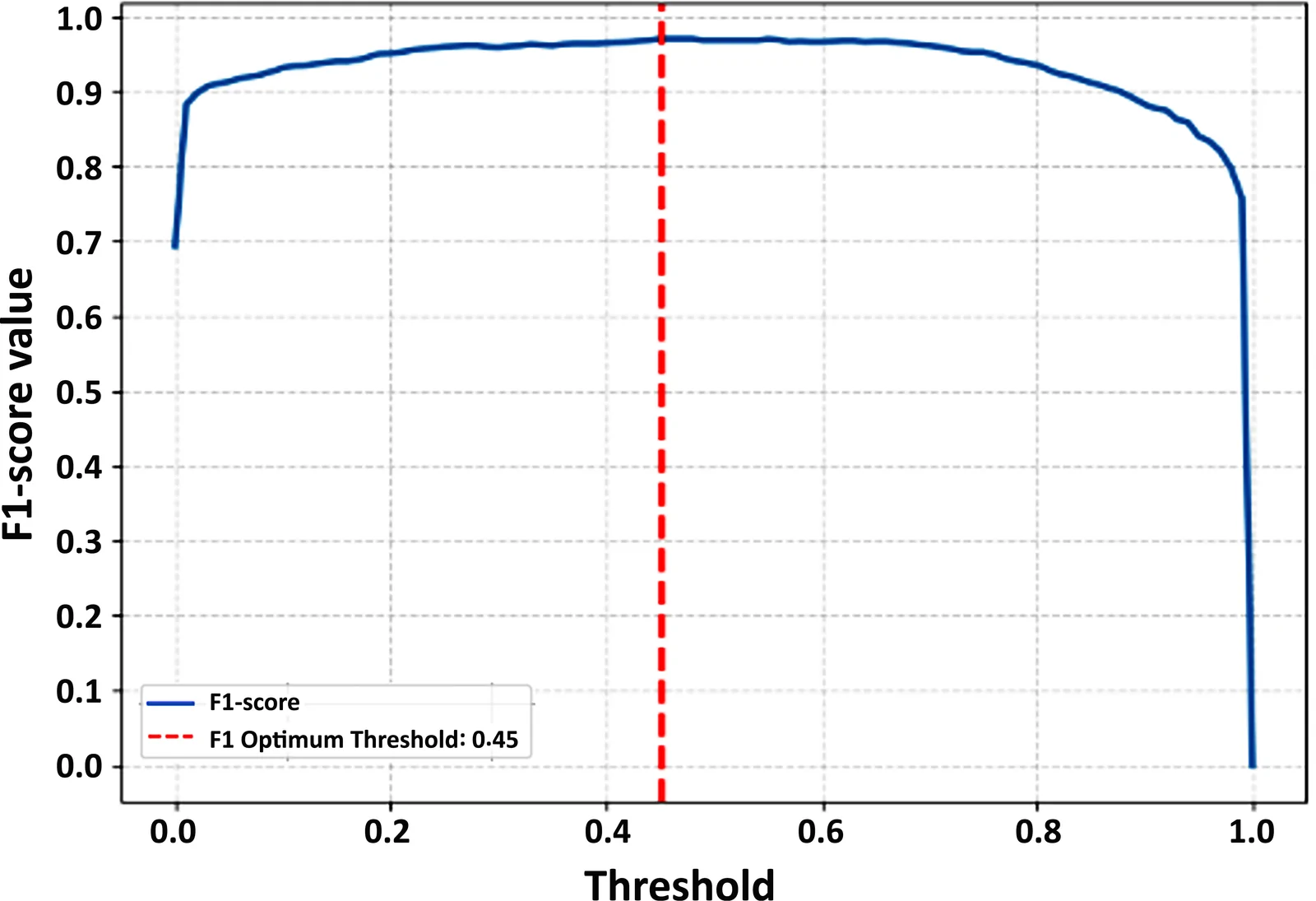
F1-score plot by classification threshold *τ_fuz_*.

Besides the classification threshold, the sigma value adjusts the “softness” of the Gaussian membership function, which determines the degree to which the model accepts a prediction as appropriate for a class. The smaller the sigma, the more stringent the fit requirement, accepting only truly “clear” examples. The larger the sigma, the more flexible the evaluation model is, accepting even “approximate” examples, but it may reduce accuracy. Figure **7** illustrates the F1-score sensitivity to the classification threshold. After determining the optimal classification threshold at 0.45, the next step is to analyze the effect of the sigma parameter in the Gaussian membership function on the performance of the model. Table **4** shows the evaluation indexes when changing value from 0.1 to 0.5, in which the Accuracy, Macro F1, and F1-score indexes for each class (Benign and Malignant).

The results in Tables **4** and **5** mention that as σ increases from 0.1 to 0.5, the model's performance tends to decrease. At σ = 0.1, the model achieves the highest Accuracy and Macro F1; however, this is too strict an evaluation threshold, only accepting very “clear” samples, easily ignoring edge cases or cases with few features. On the contrary, when σ increases to 0.3 or more, the accuracy and the overall class balance begin to decline significantly, especially at σ = 0.5, the model becomes too “soft”, reducing the ability to distinguish between two classes. Therefore, the σ value = 0.2 is chosen because it achieves good performance (Accuracy 98.77%, Macro F1 98.76%), and at the same time provides a good balance between accuracy and generalization ability. This sigma level helps the model maintain high reliability, but is still flexible enough to handle samples with less than perfect characteristics, an important factor in real-world classification problems.

The confusion matrices across all nine scenarios reveal notable variations in classification performance between the Benign (B) and Malignant (M) classes. In general, most models demonstrate a relatively high capacity to correctly identify malignant cases, as indicated by consistently high values of True Positives (TP) in the lower right quadrant. In Sce. 9, the model achieved near-perfect classification performance with 558 true positives and only 9 false negatives, highlighting its exceptional ability to detect malignant cases. Conversely, Sce. 5 and Sce. 7 exhibit comparatively weaker performance, particularly in classifying benign cases, as shown by a larger number of false positives (102 and 59, respectively). These errors suggest a model tendency toward over-predicting the malignant class, potentially due to insufficient feature discrimination. Sce. 1, 2, 3, and 8 show well-balanced confusion matrices with relatively low misclassification rates for both classes, demonstrating consistent and reliable classification ability. In particular, Scenario 8 stands out for achieving both low false-positive (20) and false-negative (16) counts, reflecting a strong trade-off between sensitivity and specificity.

Overall, the confusion matrices suggest that classification models in Sce. 2, 3, 8, and 9 perform optimally with high true positive and true negative rates.

### 5.4 Results of Detection and Classification

**Fig. (8):**
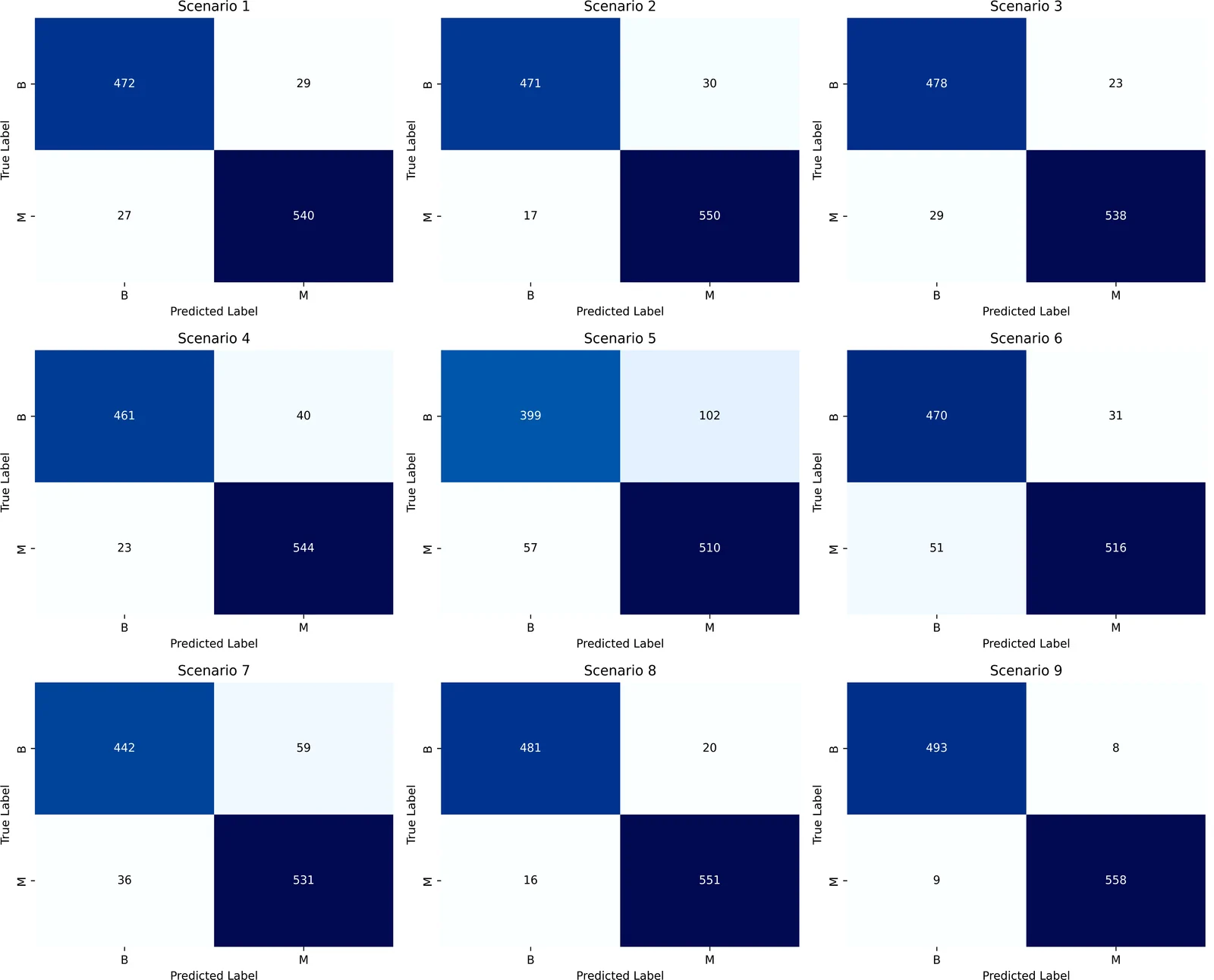
Confusion matrix between all scenarios.

**Fig. (9):**
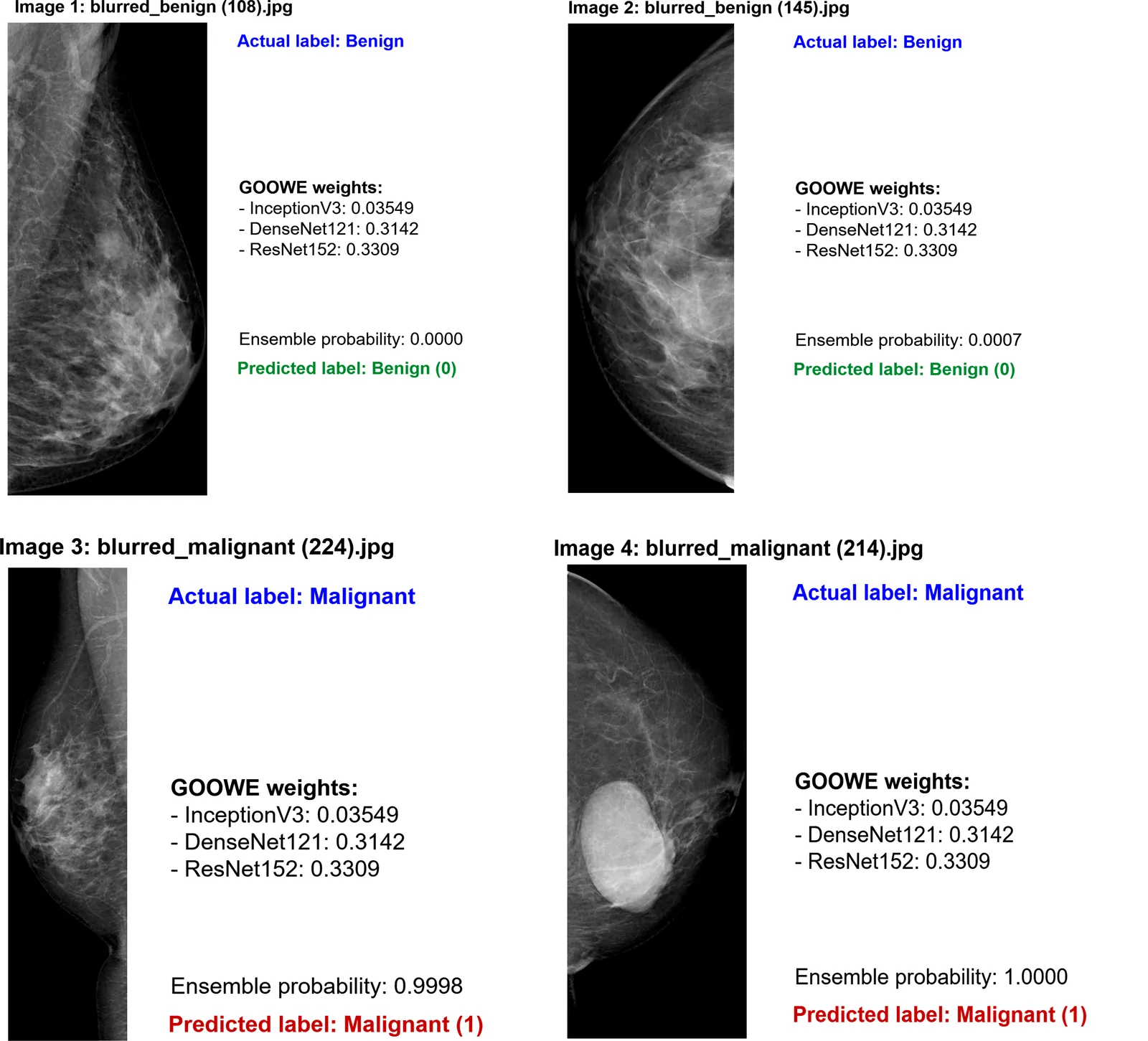
Classification results on the test set of scenario 8.

**Fig. (10):**
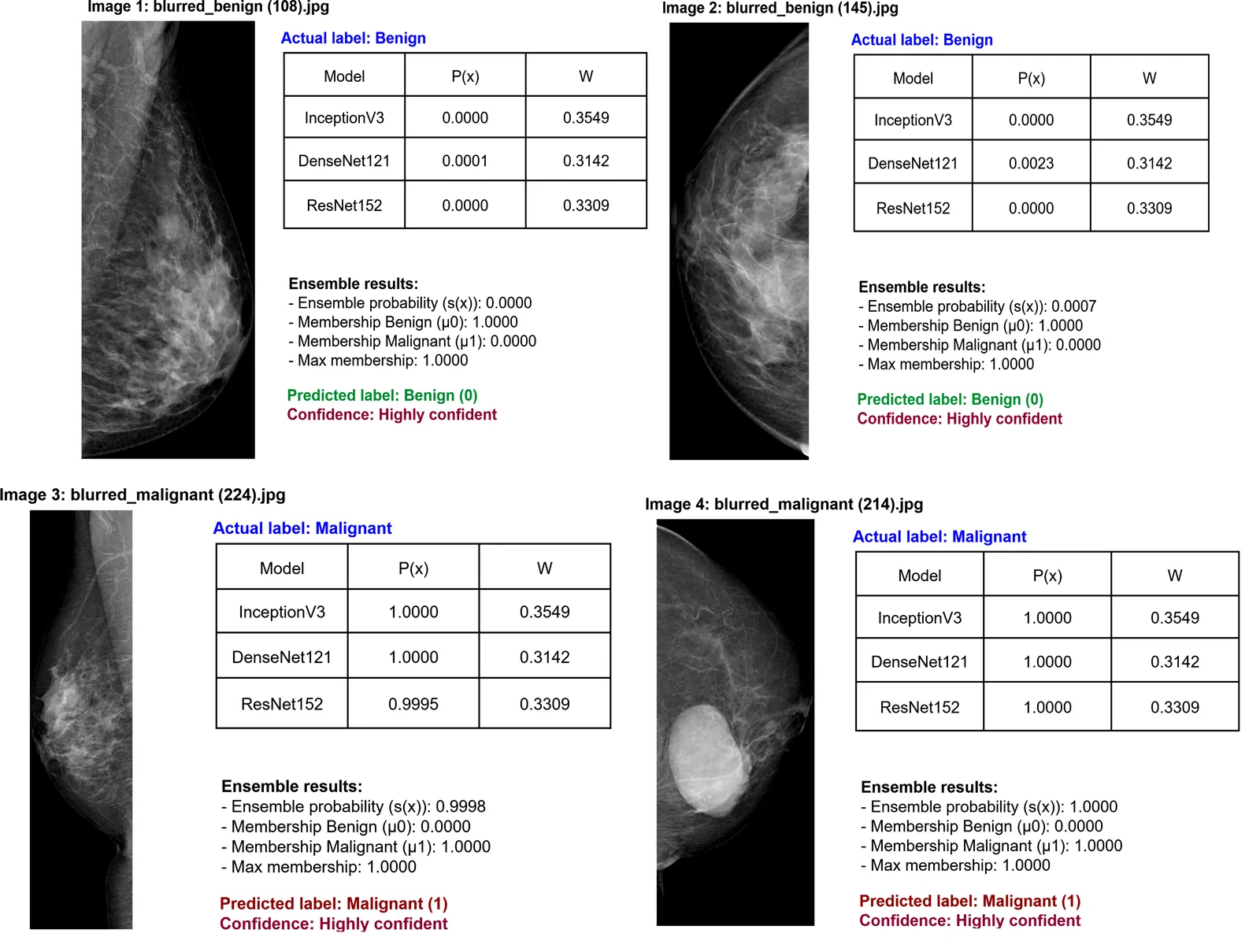
Classification results on the test set of scenario 9.

Figure **8** shows the confusion matrix and prediction results on the test dataset (Figs. **9** and **10**), applied on four breast images to the three most accurate deep learning models among the training models. The results clearly demonstrate the effectiveness of the weight optimization method in improving the accuracy and reliability of the classification process. On the test images, the ensemble model correctly predicted the Malignant label, with an ensemble probability distribution ratio of over 99%. This clearly reflects the strong recognition ability of the proposed method for malignant lesions. The ensemble results not only show the accuracy but also clearly demonstrate the high level of certainty in the decision of the ensemble model when classifying benign cases. The consistency among the models is also evident in images 3 and 4 (Malignant), where all produce consistent outputs on the input data, reinforcing the reliability of the ensemble model.

Another notable point is that the weight distribution is relatively balanced among the top three models in the ensemble of scenario 9, which is even across the high-accuracy models, specifically 30-35% across the top 3 models. The fact that no model dominates the weight distribution is a positive sign, indicating that the weight-learning mechanism in scenario 9 is not biased and has optimized the combination based on the actual performance of each component model. Due to this balance, the ensemble model remains stable, not affected by biased predictions from a single model.

## 6 DISCUSSION

This study presents FGOOPNet, a fuzzy deep ensemble model that integrates GOOWE weighting and Gaussian-based fuzzy inference to enhance mammographic breast cancer classification. The experimental results demonstrate that combining multiple complementary CNN architectures with an uncertainty-aware fuzzy reasoning layer yields significant performance improvements over individual deep models and conventional ensemble strategies.

To position our approach within the state of the art, Table **6** summarizes representative prior studies on breast cancer classification across classical ML ensembles, single CNN backbones, and advanced deep ensembles. We report the headline metric from each study for reference.

Previous works on breast cancer classification have employed diverse approaches, each with unique strengths and limitations. Traditional ensemble methods, such as majority voting over classifiers like SVM, KNN, and Random Forest [6], have shown effectiveness on balanced datasets (97.84% on BreakHis) but lack adaptability to evolving data distributions and often degrade under class imbalance. Hybrid deep learning architectures, for example, BCDNet with VGG16 [7] (94.5% on a private ultrasound dataset) and CNN with Bi-LSTM [10] (99% on MIAS), achieve high accuracy but depend heavily on feature extraction quality and incur high computational cost. Data augmentation strategies [8] can improve generalization (98% on BreakHis) but increase preprocessing complexity without addressing prediction uncertainty. EfficientNet-based designs [9] (98.23% on BreakHis) and ensemble methods integrating handcrafted or phase-based features [11] (93.47% on CSAW-M) offer competitive results but struggle with explicit uncertainty modeling and real-time adaptability. Adaptive voting strategies [17] (97.66% on WBCD) deliver incremental gains but require manual tuning and remain static after deployment. Meta-learning CNN ensembles [18] (90% on BUSI) and deep ensembles [19] (98.08% on BreakHis) enhance robustness compared to single models but still demand retraining under distribution shifts.

The proposed method addresses these challenges by integrating the Geometry-Aware Online Weighting Ensemble (GOOWE) with Gaussian fuzzy inference. GOOWE dynamically adjusts classifier weights according to real-time performance, ensuring that stronger models exert greater influence while preserving diversity. The Gaussian membership functions provide an uncertainty-aware decision layer that filters or penalizes low-confidence predictions, thereby reducing false positives and false negatives in borderline cases. Evaluated on a combined VinDr-Mammo and private expert-labeled dataset, the proposed method achieves 98.4% accuracy and delivers superior balanced class-wise F1 scores. This adaptive and uncertainty-aware framework enhances robustness, interpretability, and reliability, making it well-suited for clinical environments where data characteristics evolve over time.

### 6.1 Performance Analysis of Base Models and Ensemble Behavior

Across the nine experimental scenarios, individual CNN backbones displayed heterogeneous performance characteristics. Architectures such as InceptionV3, DenseNet-121, and DenseNet-201 consistently outperformed deeper or more computationally intensive models such as ResNet-152, indicating that extreme depth does not necessarily translate into higher accuracy for mammographic textures. This aligns with previous findings that mammogram-specific features benefit more from multi-scale feature diversity than depth alone.

The GOOWE ensemble mechanism effectively leveraged these complementary strengths by assigning higher weights to more reliable models. The ensemble results surpassed all individual CNNs by 2–7% in accuracy and produced more balanced F1-scores between benign and malignant classes. This supports the benefit of geometry-based online weighting over static voting approaches and confirms the suitability of GOOWE for handling heterogeneous feature distributions in mammography.

### 6.2 Contribution of Fuzzy Gaussian Inference to Uncertainty Management

A key contribution of this work is the incorporation of a fuzzy Gaussian inference layer, which evaluates the membership strength of the ensemble score for benign and malignant classes. This process provides a soft decision boundary that more accurately handles borderline cases—an area where classical CNNs often struggle. The fuzzy module filters low-confidence predictions using a reliability threshold, reducing both false positives and false negatives in ambiguous regions. Sensitivity analysis showed that σ = 0.2 and a threshold of 0.45 provided the optimal trade-off between precision and recall, demonstrating the practical value of incorporating uncertainty modeling into breast cancer classification.

### 6.3 Clinical Implications and Workflow Integration

From a clinical perspective, FGOOPNet offers practical benefits for radiologists and screening workflows. The ability to quantify prediction reliability using fuzzy membership degrees provides interpretable confidence indicators that can assist radiologists in assessing difficult or ambiguous mammographic regions. The system can be integrated into existing PACS/RIS pipelines as a decision-support layer. After image acquisition, the model can automatically generate malignancy scores and fuzzy confidence annotations, enabling radiologists to prioritize high-risk cases, reduce inter-observer variability, and support more consistent decision-making, particularly for dense breast cases or subtle lesions.

### 6.4 Comparison with Existing Methods

When compared to state-of-the-art breast cancer classification methods, FGOOPNet achieves competitive or superior performance. Traditional machine learning ensembles, EfficientNet-based classifiers, and hybrid CNN architectures have demonstrated strong accuracy in prior studies; however, they typically lack adaptive weighting, online learning capability, or explicit uncertainty modeling. Deep ensemble methods also require retraining under distribution shifts, whereas GOOWE provides dynamic adaptability. Moreover, few existing systems incorporate an interpretable fuzzy reasoning component. Therefore, the performance gains and interpretability benefits of FGOOPNet position it as a promising candidate for real-world deployment.

### 6.5 Limitations and Future Directions

Despite its strengths, the proposed model has certain limitations. The ensemble relies on seven CNN backbones, which increases computational complexity during training and may limit deployment in resource-constrained environments. In addition, although the hybrid dataset enhances clinical relevance, it is still limited to two sources, which may restrict generalizability across diverse imaging devices or populations. Further external validation with multi-center datasets is required to confirm robustness.

Future extensions of FGOOPNet will incorporate multi-modal imaging, such as ultrasound and MRI, to further enhance diagnostic precision. The fuzzy inference system can also be expanded by integrating additional linguistic rules or adaptive membership learning, supporting more transparent and human-interpretable explanations for radiologists. These enhancements will strengthen the clinical applicability and explainability of the model.

## 7 LIMITATIONS

Despite its promising results, FGOOPNet has several limitations that warrant attention. The ensemble relies on multiple deep architectures, which increases computational cost during training and may restrict deployment in low-resource clinical environments. Although the hybrid dataset improves generalizability, it remains limited to two imaging sources; larger, multi-institutional datasets are necessary to validate robustness across different devices, breast densities, and demographic groups.

Future work will explore adaptive or learnable fuzzy membership functions, enabling the system to refine its uncertainty modeling automatically. Expanding the framework to incorporate additional modalities, such as ultrasound and MRI, may further improve diagnostic performance, particularly for dense breasts or complex lesions. Integrating lesion localization or segmentation modules may also enhance clinical interpretability. Ultimately, large-scale prospective studies and integration within PACS/RIS systems will be essential to assess FGOOPNet’s real-world impact on clinical workflow.

## CONCLUSION

This study introduces FGOOPNet, an efficient hybrid ensemble framework designed to enhance breast cancer classification from mammography images. The method integrates the Generalized Online Weighted Ensemble (GOOWE) mechanism with Gaussian-based fuzzy inference, thereby improving predictive performance and interpretability, particularly in borderline or ambiguous cases.

A comprehensive dataset was constructed by merging the public VinDr-Mammo dataset with an additional private dataset annotated by experienced radiologists, ensuring both diversity and clinical relevance. Several state-of-the-art convolutional neural networks (InceptionV3, ResNet, DenseNet, VGG19, and Xception) were employed for feature extraction. The ensemble weights of these base models were optimized through the GOOWE approach, followed by a Gaussian fuzzy inference system to quantify prediction certainty and effectively manage uncertain classifications.

Experimental evaluation demonstrated that FGOOPNet achieved an overall accuracy of 98.4% and a balanced F1 score across benign and malignant categories, outperforming both individual deep learning models and conventional ensemble techniques. Sensitivity analysis further revealed that a confidence threshold of τ*_fuz_* = 0.45 with σ = 0.2 yielded optimal trade-offs among accuracy, sensitivity, and generalizability.

These results confirm the robustness and clinical applicability of FGOOPNet, particularly under complex data distributions and inherent diagnostic uncertainties. Future work will focus on extending the framework toward multi-stage breast cancer grading and incorporating complementary imaging modalities such as ultrasound and MRI to further enhance diagnostic reliability.

## Figures and Tables

**Fig. (2):**
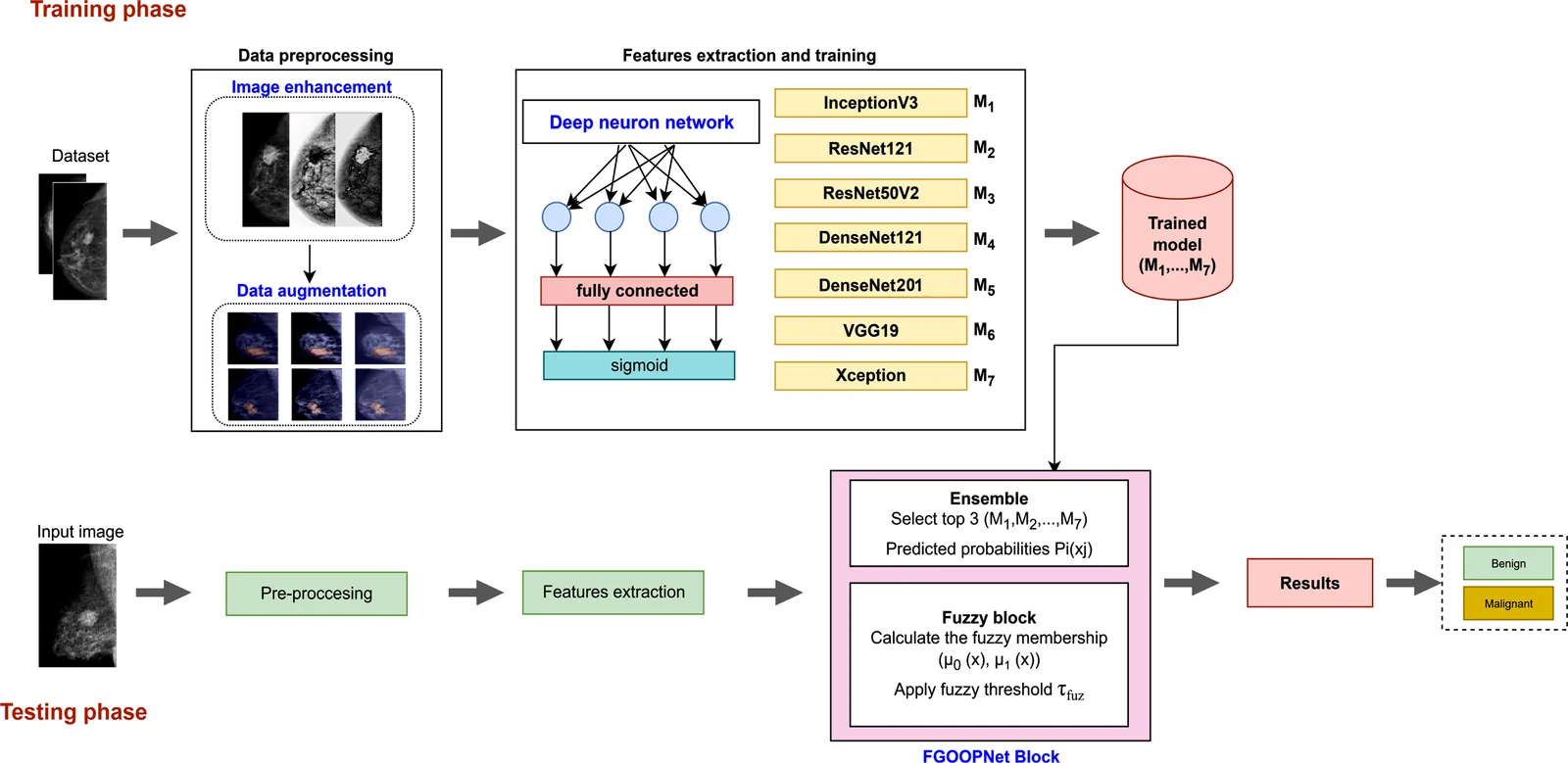
Proposed method for classifying cancer on mammograms.

**Fig. (3):**
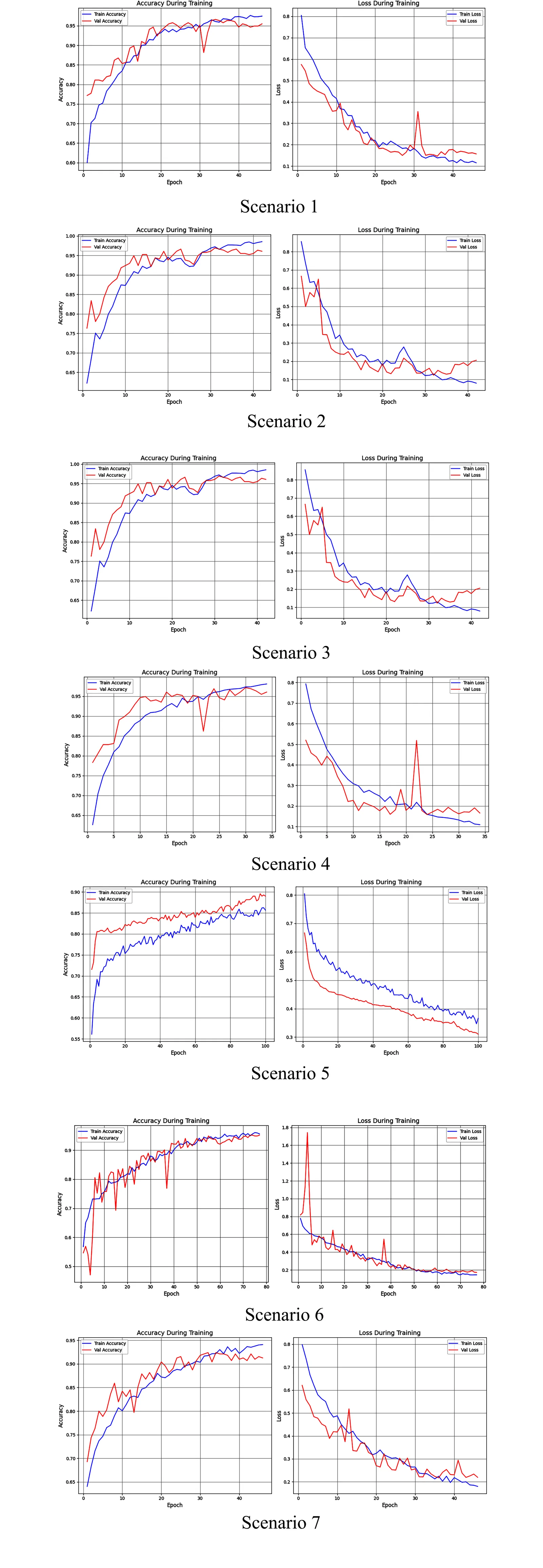
Accuracy measurement in seven scenarios, respectively.

**Fig. (6):**
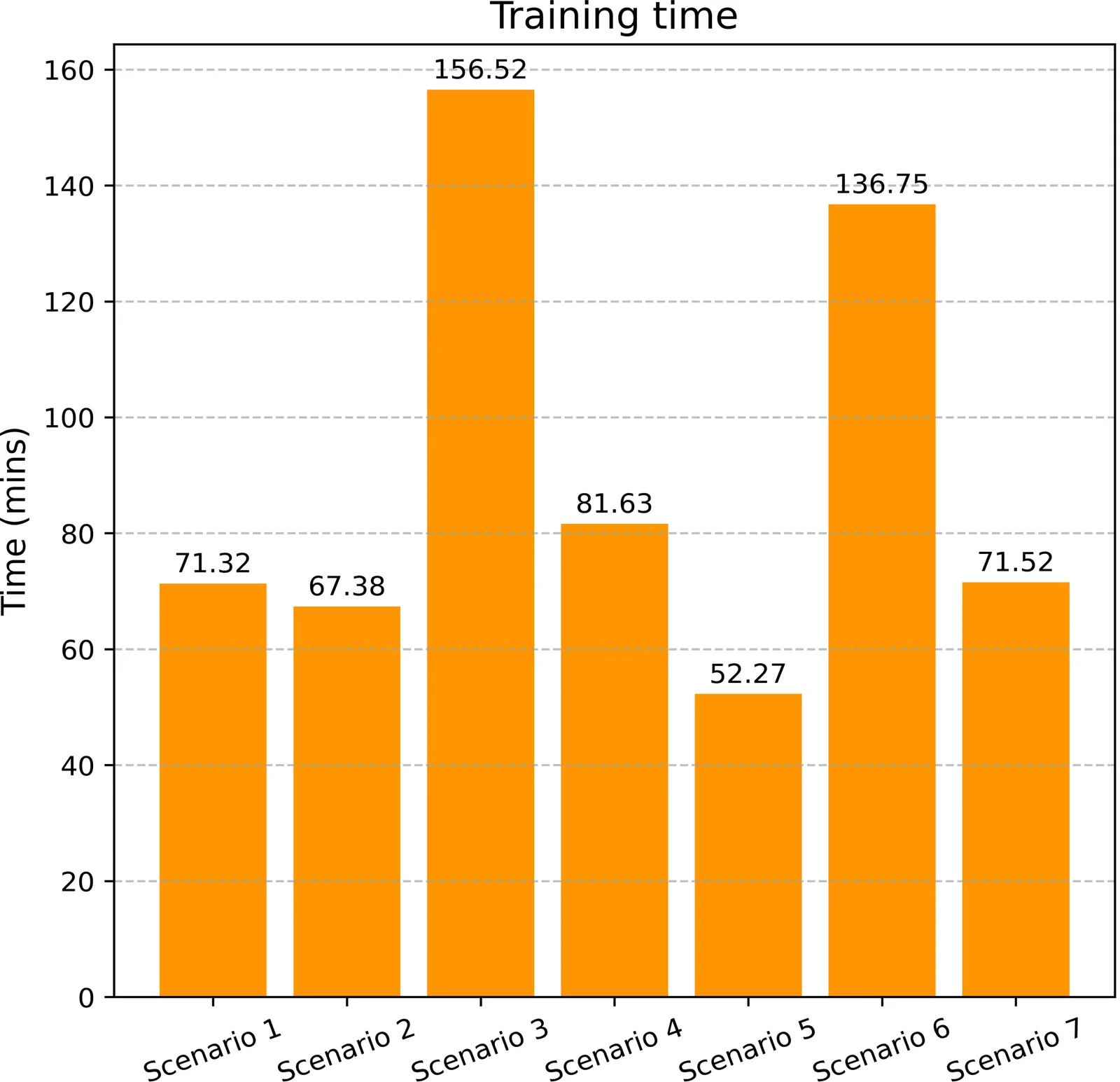
Training time between trained models.

**Table 1:** Symbols used throughout the paper.

**Symbol**	**Meaning**	**Domain/Type**	**First use**
𝑥	Input mammogram image	Image/tensor	Methodology
*ℓ*	Ground-truth class label (benign=0, malignant=1)	{0, 1}	Alg. 1
𝑦	One-hot label vector (general K classes; K=2)	{0, 1}^K^	GOOWE
𝑀	Base models (7 total; top-3 used in ensemble)	ℕ	Methodology
*C_i_*	i^th^ base classifier / CNN backbone	-	Methodology
*c*	Mean of gaussian function	ℕ	GOOWE
*A*	The matrix of predicted probabilities	Matrix	Alg. 1
*P_i_*(*x*)	Prediction probability	[0, 1]	Alg. 1
w = [w_1_, . . ., w_M_ ]	Nonnegative weights (∑*_i_w_i_* = 1)	ℝ*^M^*	GOOWE
*s*(*x*)	Weighted ensemble score (∑*_i_w_i_P_i_*(*x*))	[0, 1]	Alg. 1-2
*µ*	Center (mean) of the Gaussian membership function	{0, 1}	Alg. 2
σ	Std. of Gaussian membership (default 0.2)	ℝ_+_	Alg. 2
*µ*_0_(*x*), *µ*_1_(*x*)	Membership degrees to benign/malignant	(0, 1]	Alg. 2
τ*_cls_*	Decision threshold on *s*(*x*)	[0, 1]	Alg. 1
τ*_fuz_*	Fuzzy reliability threshold	[0, 1]	Alg. 2
TP, FP, TN, FN	Confusion-matrix counts	ℕ	Metrics
ⅅ*_train_*, ⅅ*_val_*_,_ⅅ*_test_*	Dataset splits	Sets	Data

**Table 2:** Training parameters and scenarios of the proposed method.

**Scenario**	**Model**	**Input size (HxW)**	**Batch size**	**Epochs**	**Optimizer**	**Learning Rate**
1	InceptionV3	384x192	32	100	Adam	0.0001
2	Resnet-152	384x192	32	100	Adam	0.0001
3	Resnet-50V2	384x192	32	100	Adam	0.0001
4	Densenet-121	384x192	32	100	Adam	0.0001
5	Densenet-201	384x192	32	100	Adam	0.0001
6	VGG19	384x192	32	100	Adam	0.0001
7	Xception	384x192	32	100	Adam	0.0001
8	GOOPNet					
9	FGOOPNet					

**Table 3:** Statistics of the dataset used.

**Training**	**Validation**	**Testing**	**Total**
3738	534	1068	5340

**Table 4:** Summary of model performance evaluation according to different σ values.

**σ**	**Accuracy (%)**	**Macro F1-score (%)**	**F1-score Benign (%)**	**F1-score Malignant (%)**
0.1	99.01	98.92	99.01	99.14
0.2	98.77	98.76	98.69	98.84
0.3	98.14	98.13	98.00	98.26
0.4	97.59	97.58	97.41	97.75
0.5	97.50	97.49	97.31	97.66

**Table 5:** Performance metrics for different scenarios and classes.

**Sce.**	**Class**	**Precision**	**Recall**	**F1-score**	**mAP**
1	B M	94.6 94.2	94.6 94.9	92.0 91.0	94.7
2	B M	96.5 94.8	94 97	95.3 95.9	95.6
3	B M	94.3 95.9	95.4 94.9	94.8 95.4	95.1
4	B M	95.3 93.2	92 95.9	93.6 94.5	94.1
5	B M	87.5 83.3	79.6 90	83.4 86.5	85.1
6	B M	90.2 94.3	93.8 91	92 92.6	92.3
7	B M	92.5 90	88.2 93.7	90.3 91.8	91.1
8	B M	96.78 96.5	96.01 97.18	96.39 96.84	96.63
9	B M	98.72 98.13	97.88 98.87	98.29 98.5	**98.4**

**Table 6:** Comparison with prior studies.

**Author/Refs.**	**Method**	**Dataset**	**Modality**	**Accuracy (%)**
Dhas & Singh [6]	SVM, KNN, and Random Forest	BreakHis	Histopathology	97.84
Ali *et al*. [7]	Hybrid deep learning architectures BCDNet with VGG16	Private	Ultrasound	94.5
Kaffashbashi *et al*. [8]	Data augmentation strategies	BreakHis	Histopathology	98
Behzadpour *et al*. [9]	EfficientNet	BreakHis	Histopathology	98.23
Lilhore *et al*. [10]	Hybrid deep learning architectures CNN, Bi-LSTM	MIAS	Mammogram	99
Molina *et al*. [11]	EfficientNet	CSAW-M	Mammogram	93.47
Batool & Byun [17]	Adaptive voting strategies	WBCD	Mammogram	97.66
Ali (M.D.) *et al*. [18]	Meta-learning CNN ensembles	BUSI	Ultrasound	90
Das *et al*. [19]	Deep learning ensembles	BreakHis	Histopathology	98.08
Proposed method	Fuzzy Deep Learning	VinDr + Private	Mammogram	**98.4**

## Data Availability

The VinDr-Mammo dataset is publicly available at: https://vindr.ai/datasets/mammo. The private hospital dataset used in this study is available from the corresponding author [A.P.] upon reasonable request due to patient confidentiality restrictions.

## LIST OF ABBREVIATIONS

FFDM: Full-Field Digital Mammography
CAD: Computer-Aided Detection
GOOWE: Generalized Online Weighted Ensemble
TP: True Positives
FP: False Positives
FN: False Negatives
